# Trends of Bead Counting-Based Technologies Toward the Detection of Disease-Related Biomarkers

**DOI:** 10.3389/fchem.2020.600317

**Published:** 2020-12-21

**Authors:** Wenjiao Fan, Dou Liu, Wei Ren, Chenghui Liu

**Affiliations:** ^1^Key Laboratory of Applied Surface and Colloid Chemistry Ministry of Education, Xi'an, China; ^2^Key Laboratory of Analytical Chemistry for Life Science of Shaanxi Province, Xi'an, China; ^3^School of Chemistry & Chemical Engineering, Shaanxi Normal University, Xi'an, China

**Keywords:** bead counting strategy, statistical counting, planar array, suspension array, digital counting

## Abstract

Nowadays, the biomolecular assay platforms built-up based on bead counting technologies have emerged to be powerful tools for the sensitive and high-throughput detection of disease biomarkers. In this mini-review, we classified the bead counting technologies into statistical counting platforms and digital counting platforms. The design principles, the readout strategies, as well as the pros and cons of these platforms are introduced in detail. Finally, we point out that the digital bead counting technologies will lead the future trend for the absolute quantification of critical biomarkers, and the integration of new signal amplification approaches and routine optical/clinical instruments may provide new opportunities in building-up easily accessible digital assay platforms.

## Introduction

**Scheme 1:**
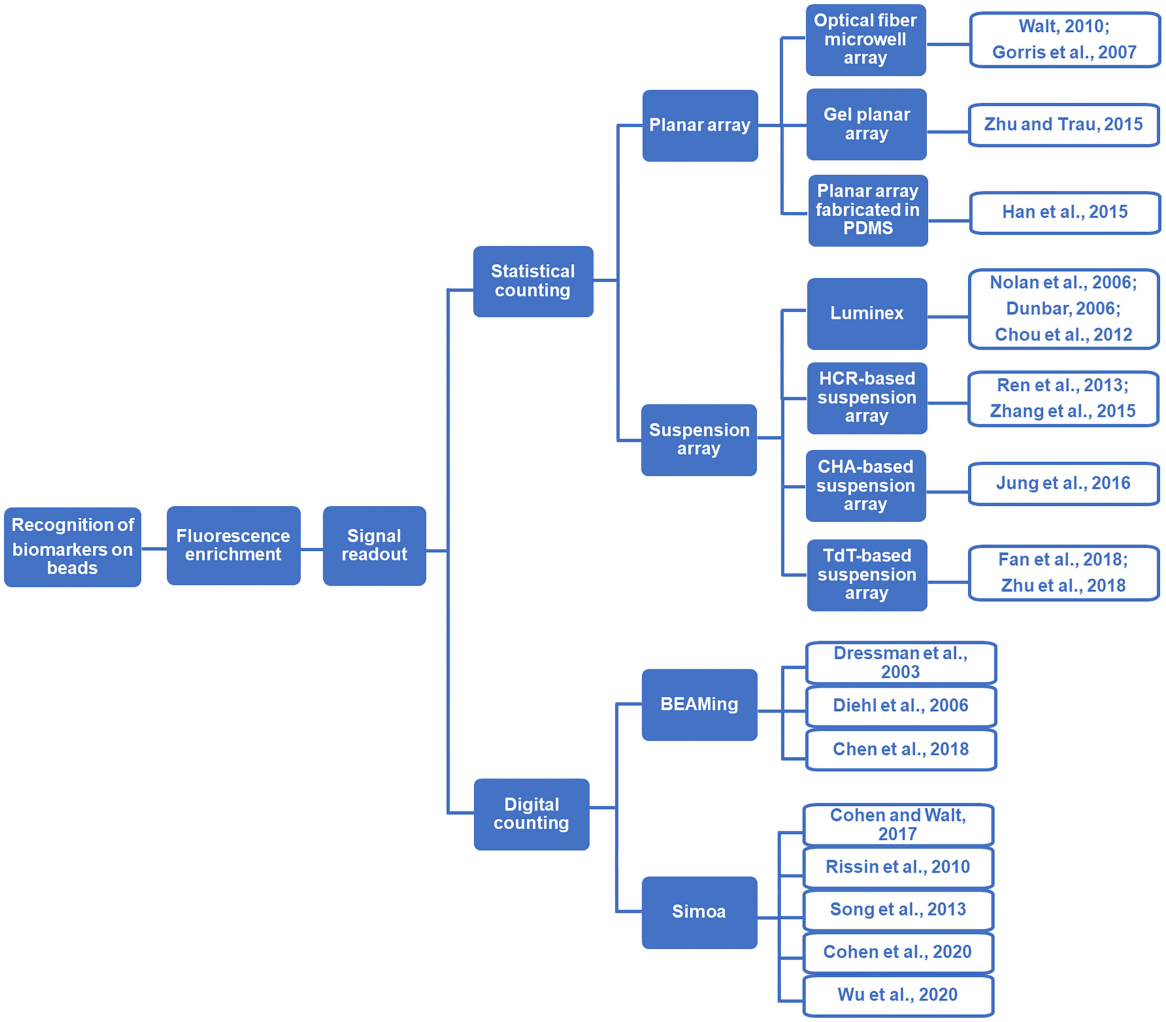
Flow diagram illustration of bead counting-based technologies.

Motivated by the growing demand for rapid and precise analysis of critical biomarkers (e.g., disease-associated nucleic acids and proteins), particle-based biomolecular assays and particle counting technologies have significantly enlarged the toolbox for bioanalysis (Rödiger et al., 2014). A lot of efforts have been devoted to the nanoparticle counting-based assays. For example, the nanoparticle counting strategies based on total internal reflection fluorescence microscopy (TIRFM) and dark field microscopy have been successfully applied for the sensitive detection of biomarkers (Ma et al., 2016, 2019; Qi et al., 2018). Compared with nanoparticles, micro-sized beads, especially magnetic beads have exhibited the potential to be adopted in broader application scenarios. In this review, we mainly focus on the particle-counting technologies which allow the precise readout of the target-induced fluorescence signals accumulated on microbeads. Generally, we classified this area into two categories (Scheme 1). One is the statistical counting, in which the target molecules are concentrated on the beads that act as the reaction carriers for fluorescent signal amplification and transduction, and the quantification of target molecules is achieved by counting/measuring the total fluorescent signals loaded on the beads. The other one is the digital counting strategy, in which each bead carries only one or none target molecule following Poisson distribution. Thus, after the single target-initiated fluorescence generation, the absolute number of the target biomolecules can be achieved by digitally counting the number of fluorescence carriers (e.g., beads). Regarding the precision and convenience of the biomolecular assays, bead counting strategies are superior to the homogeneous sensing strategies in two aspects. On the one hand, in the bead counting methods, the target biomolecule (such as nucleic acids, proteins, and antibodies/antigens)-induced response signals are anchored on the beads, which can get rid of the interference by separating the beads from the complex sample matrix. On the other hand, the commercially available and easily synthetic beads are solid and stable, which can be applied as satisfying microreactors for multi-step reactions. For example, compared with the emulsions used as microreactors in classical digital assays (e.g., droplet digital PCR, abbreviated as ddPCR), the bead-based digital assays are steady and facile due to the avoidance of using fragile emulsion droplets. Therefore, it is believed that the prosperity of the bead-counting technologies will lead to the more precise detection of critical biomarkers.

## Beads-Based Strategies for the Detection of Biomolecules

### Beads-Based Statistical Counting Strategies for the Detection of Biomolecules

In the statistical counting strategies, the fluorescence signals are accumulated on the beads via target-induced reactions. Therefore, the fluorescence intensity statistically collected from a large population of beads can reflect the concentration information of the target molecules. It should be noted that in such strategies, the concentration of target can only be obtained by substituting the experimental data into the standard curve established by a gradient of standard samples with known concentrations. Basically, there are two representative types of statistical bead counting strategies: the solid-state planar arrays and the liquid-state suspension arrays, both of which have been applied widely in the detection of disease-related biomolecules (Parsa et al., 2018; Vafajoo et al., 2018). In the solid-state planar arrays, the beads are attached to a solid substrate after target capturing and fluorescence signal accumulation, and the fluorescence signals of the beads are monitored after the washing procedure (Sukhanova and Nabiev, 2008). As to the suspension arrays, after introducing the target-associate fluorescent tags onto the beads, the beads are monitored one by one when they pass through the laser beam without any separation procedure (Leng et al., 2015).

#### Solid-State Planar Bead Arrays

Generally, in the planar bead arrays, the target-specific fluorescence reporters are immobilized on the beads, which are then deposited onto a solid substrate or into microwells (Rödiger et al., 2014). Then, the beads carrying fluorescent signals are captured using an imaging system (e.g., a fluorescence microscope). In the following steps, the fluorescence images are processed and annotated by professional software for target quantification.

One of the most prominent planar bead strategies is the optical fiber microwell array described by Walt's group (Gorris et al., 2007; Walt, 2010). In this working principle, the target-specific beads are distributed into the microwells generated by hydrofluoric acid etching on an optical imaging fiber. After removing the extra beads and solutions, the fluorescence intensity of the beads is collected by an imaging system. Therefore, the amount of target can be reflected by the fluorescence signals specifically accumulated on the beads. Benefiting from the versatile design, not only nucleic acid but also protein can be sensitively analyzed with this array.

Zhu and Trau (2015) presented a gel planar array chip for high-throughput and multi-analyte bead-based immunoassays. The chip is fabricated on a glass slide by using polyacrylamide gel and polyethylene glycol (PEG) gel. The resulting chip consists of a number of polyacrylamide gel units for the immobilization of beads, and each gel unit is surrounded with a PEG ring to confine the sample within the microarrays. Consequently, the on-bead immunoreaction was confined in the microarrays. After the immunoreaction with the Alexa Flour labeled detection antibody, the target-specific fluorescent beads are monitored with a fluorescence microscope, and the target can be quantified by recording the total fluorescence signal loaded on the beads. In this method, the detection limits below the physiological threshold level for cancer diagnosis was achieved.

The Koh group developed a microhole planar array fabricated in PDMS (poly-dimethylsiloxane), where each microhole was designed to trap a single bead that functionalized with probe antibodies (Han et al., 2015). The beads were coded with quantum dots (QDs) of two different colors. After the specific immunoreactions on the surface of the QDs-encoded beads, multiple target proteins can be recognized by identifying the beads in the microholes with their precision x and y coordinates recorded, and then quantified by analyzing the fluorescence signals of QDs-embedded beads with photolithography. In this design, as low as 1 ng/mL target can be detected.

#### Liquid-State Suspension Bead Arrays

Although planar arrays play an important role in ultrasensitive bioanalysis, limitations on the quality of its results, binding rates, decoding speed, and overall flexibility still exist (Wilson et al., 2006; Leng et al., 2015). Fortunately, a series of liquid-state suspension bead assay strategies relying on monitoring free beads have been proposed for the efficient analysis of biomarkers, which may solve the challenges. Typically, the flow cytometric methods that count the suspending beads carrying varied fluorescence signals individually seems to be the most dominant. Flow cytometric is a versatile technology for the rapid interrogation of individual cells or beads in a one-by-one fashion (Adan et al., 2017). It can simultaneously measure the light scattering and the fluorescence intensity of individual beads in a fluid stream when they pass through the laser beam (Wilkerson, 2012). By statistically processing the fluorescence data collected from the suspension beads, the target biomarkers that induce the fluorescence accumulation on the beads can be quantified. This working scheme makes the flow cytometer (FCM) a powerful tool for the analysis of disease biomarkers in biomedical research and clinical diagnosis.

One of the most commonly used FCM systems is the Luminex family, a well-established platform using multiple kinds of fluorophores to encode a panel of beads (~5.5 μm polystyrene beads) (Nolan et al., 2006). In this system, each bead group corresponds to a specific target biomolecule. With more than one solid-state laser equipped on the FCM, the beads with different fluorescence colors and intensities are counted one by one in the liquid flow and decoded by the detectors. In this way, multiple targets can be analyzed simultaneously by using fluorescence-encoded beads (Dunbar, 2006; Chou et al., 2012).

Despite the high-throughput monitoring of disease biomarkers, higher detection sensitivity is always desired in the clinical diagnosis. In this regard, a series of on-bead signal amplification strategies have been reported to improve the sensitivity of the liquid-state suspension bead-based assays. For example, hybridization chain reaction (HCR) (Dirks and Pierce, 2004) is one of the most effective enzyme-free amplification strategies that have been integrated with the liquid-state bead-based sensitive detection of biomarkers (Ren et al., 2013). In these designs, the mute trigger of HCR is anchored on the beads. Only in the presence of target molecules, the trigger can be activated to initiate a cascade hybridization reaction of two metastable hairpin probes (Zhang et al., 2015). By labeling the fluorophore molecules on the hairpin probes, a lot of nicked double-stranded DNA (dsDNA) structures with fluorescent signals are enriched on the beads. With the help of the efficient HCR, even a low concentration of target molecule can induce an observable fluorescence signal that can be sensed and quantitively analyzed by FCM. Catalyzed hairpin assembly (CHA) is another mature nonenzymatic nucleic acid amplification strategy (Yin et al., 2008; Li et al., 2011). In CHA, a single-stranded DNA (ssDNA) is required as the catalyst to trigger the strand exchange reactions of two hairpin probes and initiate the cycling of CHA circuits. In greater detail, the ssDNA catalyst can interact with a toehold on one of the hairpin probes (H1) and open the hairpin to expose a new ssDNA. And the newly exposed ssDNA can hybridize with a toehold on another hairpin probe (H2) and trigger the strand-exchange process. With the displacement, the free catalyst can participate in subsequent reaction cycles. In this way, lots of H1:H2 duplexes are formed even at the low level of target concentration without using the enzymes. As an enzyme-free strategy, the efficient CHA can be integrated with the bead-FCM system for the sensitive detection of biomarkers (Jung et al., 2016). And the system could be of use in analytical and diagnostic applications.

Compared with the enzyme-free amplification methods, the enzyme-involved amplification strategies are more effective. Terminal deoxynucleotidyl transferase (TdT) is a template-independent DNA polymerase that catalyzes the repetitive sequential addition of deoxynucleotides (dNTP) at the 3′-OH group of a DNA (Liu et al., 2014; Wang et al., 2017). Taking benefit of this intriguing property of TdT, effective signal amplification can be achieved without complicated probe design, which greatly simplifies the assay. Considering this, a set of high-sensitive strategies have been reported for both nucleic acid and protein sensing by conducting the target molecule-initiated TdT extension on the surface of beads (Fan et al., 2018; Zhu et al., 2018). After immobilizing fluorescent molecules on the product of target-initiated TdT extension, the beads with different fluorescence intensity are interrogated, and then statistically analyzed by FCM. In this way, as low as 5 fM nucleic acid and 0.5 pg/mL protein can be analyzed.

In conclusion, both the solid-state planar bead arrays and liquid-state suspension bead arrays can provide satisfying biomarker analyzing performance. To further improve the sensitivity of the bead-based statistical counting strategies, developing more efficient signal amplification methods and applying more sensitive fluorescence instruments are two most promising ways.

### Beads-Based Digital Counting Strategies for the Detection of Disease Biomarkers

The statistical bead counting strategies have allowed the sensitive and multiplex detection of disease-related biomolecules, however, a standard curve is inevitable. In this consideration, the most precise and promising way of quantifying the biomolecules is to count their absolute number in a digital manner (Walt, 2013; Gooding and Gaus, 2016). In digital bioassays, the sample solution containing target molecules is divided into a great deal of separate microreactors. According to the Poisson distribution, the microreactors will be of one or none target biomolecules and ultimately show either positive or negative binary signal readout (Zhang and Noji, 2017). In this way, the target biomolecules can be digitally reflected by the number of positive microreactors. Compared with the statistical counting assays, the digital counting bioassays are able to provide the absolute number of target molecules without using a standard curve, expelling a series of interfering factors. Due to such advantages, the sensitivity of digital counting bioassay is reported to be much higher than that of the statistical counting assays.

#### BEAMing Platform

**Figure 1:**
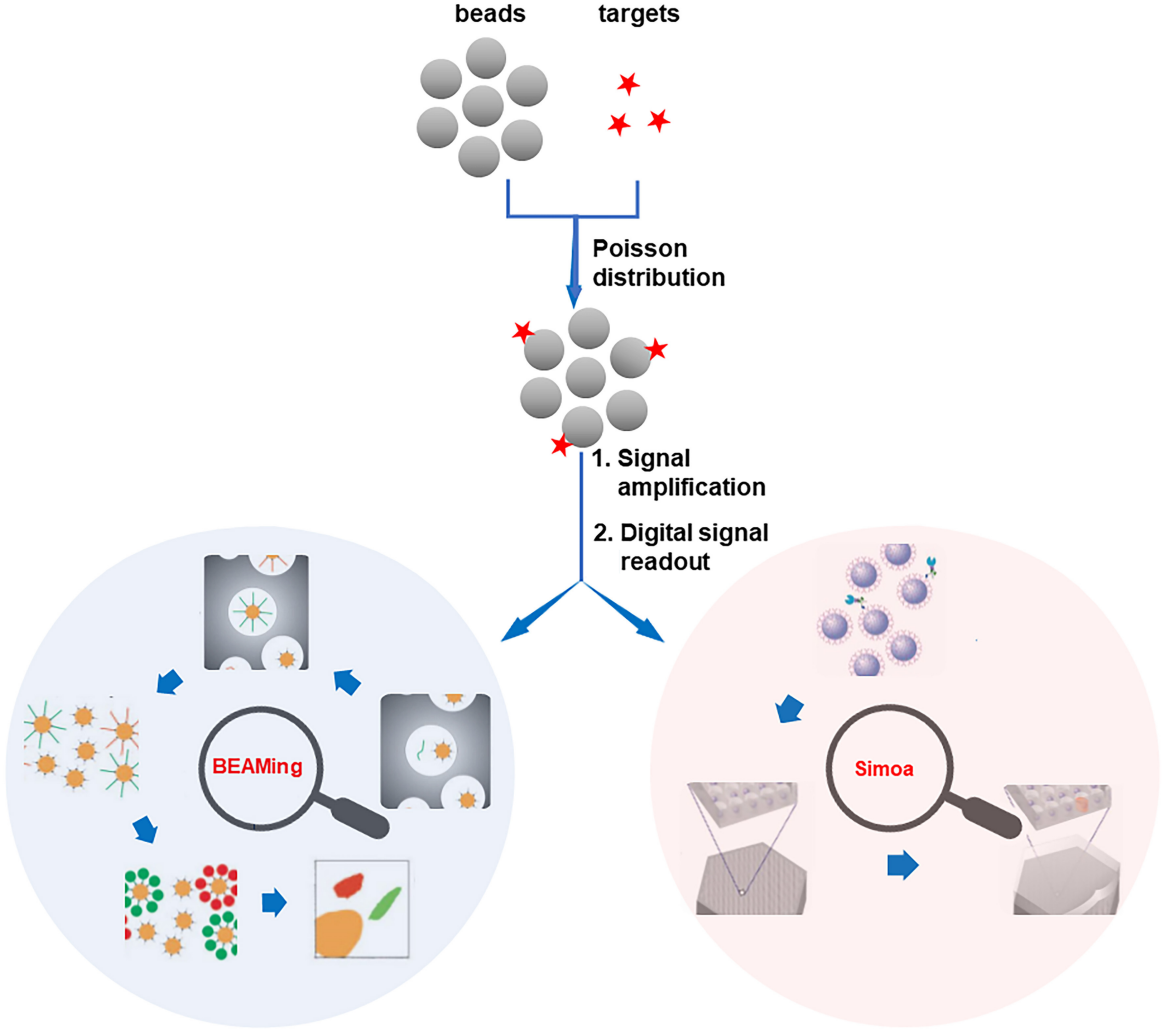
Schematic illustration of 2-well-recognized digital bead counting bioassay technologies. Reproduced with permission from Dressman et al. (2003). Copyright 2003, National Academy of Sciences (BEAMing). Reproduced with permission from Rissin et al. (2010). Copyright 2010, Nature Publishing Group (Simoa).

The ddPCR (e.g., Bio-Rad QX-100/QX-200) is the most mature digital counting bioassay, which has already been commercialized (Hindson et al., 2011; Pinheiro et al., 2012). It enables the absolute digital quantification of target molecules without the requirement of external/internal standard and is extremely suitable for precise quantification of low-abundance nucleic acids. Different from ddPCR which adopts pure emulsion droplets as the independent microreactors, BEAMing (Dressman et al., 2003) is developed depending on four principal components (beads, emulsion, amplification, magnetics, Figure 1). In BEAMing, a single target molecule and a single magnetic bead are simultaneously encapsulated in one drop of emulsion (Diehl et al., 2006; Chen et al., 2018). After the polymerase chain reaction (PCR) or loop-mediated isothermal amplification (LAMP) process on the beads inside the emulsions, the emulsions are broken and the beads are purified with the assistance of magnetic separation. After then, the number of variant DNA molecules in the population is analyzed by counting the fluorescence-positive beads using FCM. Nevertheless, in the principle of BEAMing, only the microemulsions containing both a target molecule and a bead can lead to the positive signal readout. Therefore, false negatives may occur when the nucleic acid target and bead are not encapsulated in one emulsion simultaneously. It should also be noted that the fragile emulsion reaction is prone to be affected by many factors to bring misleading results.

#### Single Molecule Arrays (Simoa)

Another prominent digital platform is the Simoa, which was firstly proposed by the Walt group (Rissin et al., 2010; Cohen and Walt, 2017). In the principle of Simoa, individual beads (~2.7 μm paramagnetic beads) bound with either none or one target molecule are seeded into femtoliter-volume well arrays (Figure 1). Assisted by an enzyme-catalyzed fluorogenic reaction, the detection of the target is converted to counting the number of positive microwells that contain the beads with the target molecule (Song et al., 2013). As a result, the subfemtomolar level of target biomolecules can be detected by the Simoa system. However, the sensitivity of Simoa is impeded by the low loading efficiency of beads, in which just about 5% of the total beads can be sampled into the microwells and analyzed (Cohen et al., 2020). To overcome this barrier, recently, the dropcast Simoa (dSimoa) system for the ultrasensitive protein detection by single molecule counting with a higher sampling efficiency is proposed (Wu et al., 2020). In this approach, instead of loading the beads into the microwells, the beads are simply dropcast onto a microscope slide for single molecule counting. Therefore, the sampling efficiency is dramatically improved.

As a conclusion, the ultrahigh sensitive quantification of disease-biomolecules can be achieved by introducing bead-based digital counting strategies, which is with great promise to offer even the absolute number of target molecules in complex biological samples. Therefore, compared with the statistical counting strategies, the bead-based digital counting strategies are more precise and sensitive.

## Perspective

**Figure 2:**
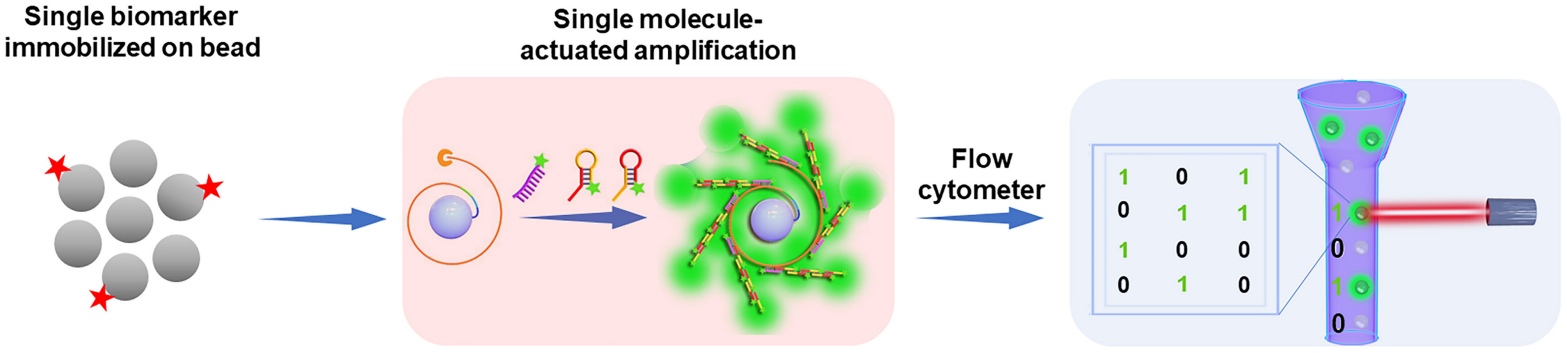
Perspective on the digital counting platform: the integration of single molecule-initiated signal amplification strategies and conventional instruments (e.g., flow cytometer) may lead the future trend of digital assay technologies. Reproduced with permission from Fan et al. (2020). Copyright 2020, The Royal Society of Chemistry.

Although the digital assay strategies, including ddPCR, BEAMing, and Simoa have shown the future trend of biomarker analysis, we anticipate that the development of new bead-based digital platforms with commonly accessible equipment, simple operation, and wide applicability is of great significance for the prevalence of digital assays. Considering the advantages of bead-based approaches and the superior of digital counting assays, our group has developed a new digital platform based on the single molecule-initiated signal amplification that can illuminate a single bead for the ultrasensitive detection of biomolecules (dFlowSeim, Fan et al., 2020). In this scheme (Figure 2), according to the principle of the digital assay, one or none target molecule is immobilized on each bead (~2.8 μm paramagnetic beads), and then an on-bead single molecule-actuated nucleic acid amplification is rationally designed to make sure that sufficient numbers of fluorescent molecules are gathered on the single bead to make it bright enough for FCM or fluorescence microscope sensing. As a result, the beads carrying one target will be identified as positive ones while the beads without capturing a target are identified as negative. Then the number of positive and negative beads can be facilely counted by the versatile FCM or fluorescence microscope to achieve the digital analysis of target biomolecules. The digital platform based on the single molecule-initiated signal amplification-illuminated beads has wide applicability. In the first place, FCM and fluorescence microscope are widely equipped and easily accessible in hospitals and ordinary biolabs. Then, this bead-based digital platform is emulsion-free, so unlike the BEAMing system, the digital analysis of target biomolecules can be achieved without the complicated emulsion-generation and emulsion-broken procedures. Additionally, it is a promising approach to achieve multiplexed biomolecule detection by encoding each kind of target molecules in a digital manner because the FCM is able to clearly discriminate the beads of varied sizes. As to the proposed digital platform, the efficiency of single molecule-initiated signal amplification is the key point for the enrichment of sufficient fluorescent molecules to illuminate the single bead. Therefore, it is predicted that with the development of high-efficient on-bead signal amplification strategies, the sensitivity, applicability, and flexibility of the bead-based digital platform will be significantly improved.

## Conclusion

In this mini-review, a series of bead counting strategies, including statistical counting and digital counting for the analysis of disease-related biomolecules have been summarized. The design principles and advantages of the most popular bead counting-based assays have been described and discussed in detail. Finally, we point out that the digital assay will lead the future trend of the bead counting-based bioassays and the integration of routine instruments and emerging on-bead signal amplification technologies will broaden the way of digital assays.

## Author Contributions

WF and DL wrote and revised the manuscript. WR supervised and revised the manuscript. WR and CL were in charge of the whole manuscript. CL put forward the central idea of the manuscript and gives final modification. All authors contributed to the article and approved the submitted version.

## Conflict of Interest

The authors declare that the research was conducted in the absence of any commercial or financial relationships that could be construed as a potential conflict of interest.
